# *Babesia lengau* associated with cerebral and haemolytic babesiosis in two domestic cats

**DOI:** 10.1186/1756-3305-6-128

**Published:** 2013-05-01

**Authors:** Anna-Mari Bosman, Marinda C Oosthuizen, Estelle H Venter, Johan CA Steyl, Tertius A Gous, Barend L Penzhorn

**Affiliations:** 1 Department of Veterinary Tropical Diseases, Faculty of Veterinary Science, University of Pretoria, Private Bag X04, Onderstepoort 0110, South Africa; 2 Department of Paraclinical Sciences, Faculty of Veterinary Science, University of Pretoria, Private Bag X04, Onderstepoort 0110, South Africa; 3 Specialist Veterinary Pathologist, P.O. Box 5371, Helderberg 7135, South Africa

**Keywords:** Babesia lengau, Cerebral babesiosis, Feline babesiosis, Haemolytic anaemia

## Abstract

**Background:**

Although reported sporadically from various countries, feline babesiosis appears to be a significant clinical entity only in South Africa, where *Babesia felis* is usually incriminated as the causative agent. *Babesia lengau*, recently described from asymptomatic cheetahs, has now possibly been incriminated as the causative agent in two severe clinical cases in domestic cats.

**Findings:**

Both cats were euthanised *in extremis*. While typical feline babesiosis in South Africa is an afebrile disease with a chronic manifestation, there was acute onset of severe clinical signs in both cats and their body temperatures were above the normal range when they were presented for treatment. Haemolytic anaemia was confirmed in one case. To our knowledge, this is the first report of cerebral babesiosis in cats.

On reverse line blot 18S rDNA PCR products obtained from both cats showed positive hybridization profiles with the *B. lengau* species-specific probe. The two partial parasite 18S rRNA gene sequences obtained, showed high sequence similarity (99.9%) to *B. lengau*. In a representative tree constructed by the neighbor-joining method using the two-parameter model of Kimura the two obtained partial 18S rDNA sequences and that of *B. lengau* formed a monophyletic group with *B. conradae* and sequences previously isolated from humans and wildlife in the western USA.

**Conclusion:**

All clinical cases of feline babesiosis in South Africa are not necessarily caused by *B. felis*. Other piroplasms, e.g. *B. lengau*, may be incriminated in clinical cases, especially those occurring outside the known endemic area.

## Findings

**Figure 1:**
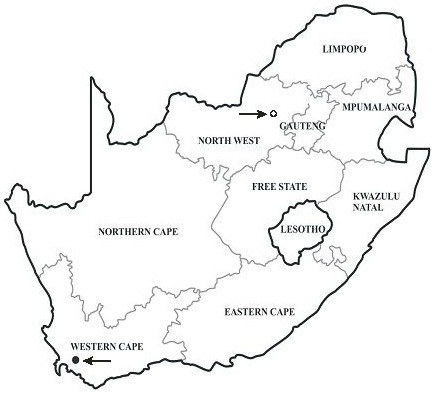
**Map of South Africa, showing the localities of the two cases.** The feline babesiosis endemic area extends along the eastern and southern coast (KwaZulu-Natal, Eastern and Western Cape Provinces), as well as along the eastern escarpment in Mpumalanga and Limpopo Provinces in the north-eastern part of the country. Case 1 (Rustenburg): Open circle. Care 2 (Wellington): Black circle.

Babesiosis of domestic cats has been reported sporadically from various countries, including France [1], Germany [2], India [3], Israel [4], Poland [5], Portugal [6], Thailand [7] and Zimbabwe [8], but appears to be a significant clinical entity only in South Africa where it occurs primarily along the eastern and southern seaboard (KwaZulu-Natal, Eastern and Western Cape Provinces), as well as in isolated foci along the eastern escarpment of Mpumalanga and Limpopo Provinces [9-11] (Figure 1). This would suggest that the vector(s) only occur(s) in these areas, but the vector(s) remain(s) unknown. Clinical cases occurring inland, e.g. in Gauteng Province, usually concern pets taken along when the owners visited the coast [9,12]. Feline babesiosis has been well documented in South Africa. Apart from case reports [13], there have been detailed studies on signalment, clinical manifestation and pathology [9,14,15] as well as treatment [12,16]. Lethargy, anorexia and anaemia generally occur, while icterus is only occasionally seen [14]. Elevated body temperature is not a feature of this disease [14]. In clinical cases, parasitaemia is usually high and may exceed 50% [14].

*Babesia felis*, the piroplasm usually incriminated as causing feline babesiosis in South Africa, was initially described from a Sudanese wild cat (*Felis ocreata*, syn. *F. sylvestris*) [17]. Fourteen domestic cats inoculated with blood from the wild cat became persistently infected, with parasitaemias < 1%, but did not develop any clinical signs [17]. When feline babesiosis was first described in South Africa a few years later, the parasite was considered to be morphologically similar to *B*. *felis*, but due to its pathogenicity in domestic cats it was called *Nuttallia felis* var. *domestica*[13]. It remains unresolved whether the pathogenic parasite in South Africa is indeed conspecific with the one described from Sudan [17].

Although the famous Kenyan lioness "Elsa" was reported to have died of babesiosis [18], *Babesia felis* sensu stricto has not been incriminated in causing disease in felids other than domestic cats. It has been reported from 18 out of 97 clinically normal captive cheetahs (*Acinonyx jubatus*) in South Africa and 3 out of 40 free-ranging cheetahs in Namibia, as well as from a single lion (*Panthera leo*) and serval (*Leptailurus serval*) in South Africa [19]. *Babesia leo*, commonly found in lions in South Africa, has also been reported from a clinically healthy domestic cat [19,20].

Recently, *Babesia lengau* was described from clinically healthy cheetahs [21]. In this retrospective study, we report two severe clinical cases of feline babesiosis in domestic cats associated with *B. lengau* infection. As far as we could ascertain, this is the first report of cerebral babesiosis in a domestic cat. In both instances, which occurred before the formal description of *B. lengau*, we did not have access to the clinical cases but interpreted necropsy reports. Both cats had been euthanised *in extremis*. One case was submitted for necropsy and nucleic acid-based diagnostics to the Faculty of Veterinary Science, Onderstepoort, South Africa. Blood specimens from the second case were submitted to confirm babesiosis. The two cases occurred in different parts of South Africa (North West Province and Western Cape Province, the latter within the known endemic area of feline babesiosis) and there was no connection between them. The results showed the presence of a *Babesia* parasite very close or similar to *B. lengau*, recently described from asymptomatic cheetahs*.*

### Case 1 (Rustenburg, North West Province; June 2004)

An entire, 2-year-old Siamese tomcat, which had been eating and playing in the morning, was presented at a veterinary practice in a collapsed state that evening. He had a temperature of 40.3°C with pale-icteric mucous membranes. Blood smears revealed intraerythrocytic protozoan parasites morphologically resembling *Babesia* species, as well as erythrocytes phagocytosed by leukocytes. The cat was euthanised and the frozen carcass was submitted for necropsy to the Pathology Section, Faculty of Veterinary Science, University of Pretoria.

Macroscopic findings were as follows: The overall body condition of the animal was very good. External examination revealed moderate flea infestation and marked icteric mucous membranes. No ticks were found on the body of the animal. On evisceration, pronounced icterus was observed, associated with watery blood (indicative of severe anaemia) on blood vessel incision. Anaemia was initially masked by severe icterus. Mild hydrothorax and hydropericardium as well as acute, centrilobular hepatosis were observed. Moderate diffuse splenomegaly due to red pulp hyperplasia and mild, diffuse pulmonary congestion and oedema occurred. On cytological examination, a splenic impression smear showed numerous free-lying groups / aggregates of spherically shaped piroplasm-like protozoan organisms among leukocytes and lytic erythrocytes. Occasional intact erythrocytes contained the same organism resembling a *Babesia* or *Theileria* species. Freeze artefacts may have influenced parasite morphology.

The histopathological conclusion was that this animal had suffered from severe prehepatic icterus and anaemia, which appeared to have resulted from a combination of intra- and extravascular haemolysis. Freeze artefacts had a negative impact on examination and interpretation.

### Case 2 (Wellington, Western Cape Province; July 2007)

An 18-month-old domestic short-haired cat was presented to a private veterinarian with a complaint of weakness. It was in good condition, had a temperature of 40.2°C and showed anisocoria. There was mild lameness of the left hind limb, which appeared to be more of extensor rigidity. There was severe anaemia and icterus, and the cat was covered in large numbers of ticks. A blood smear revealed large numbers of white blood cells, as a result of lymphocytosis, but no blood parasites were observed by the attending veterinarian. The smear also showed evidence of a non-regenerative anaemia. Treatment consisted of intravenous fluids (Ringer’s lactate) and injections of 0.3 ml dexamethasone, 0.4 ml amoxycillin and clavulanic acid (Synulox) and 0.4 ml Vitamin B Complex. The cat did show some appetite but was force-fed. During the evening nervous signs such as paddling, vocalisation and clonic spasms of the neck developed and the cat was euthanised by intravenous administration of an overdose of barbiturates.

**Figure 2:**
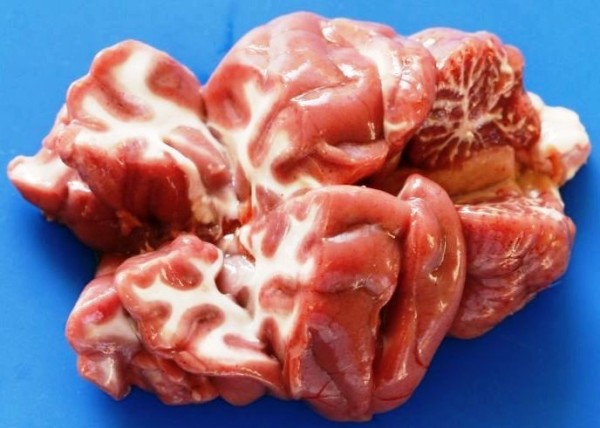
The brain of the cat (Case 2): The entire brain shows pronounced congestion with clear red-pink discoloration of the grey matter, as well as multifocal petechiae (Photograph: Tertius Gous).

A necropsy was performed by a private diagnostic pathologist. The carcass was in fairly good condition. There was marked anaemia and icterus. The entire brain showed very pronounced congestion with clear red-pink discoloration of the grey matter, as well as multifocal petechiae (Figure 2). There was severe congestion and oedema of the lungs. The pericardium contained a moderate amount of serosanguineous fluid. Moderate congestion was noted in the spleen, mesenteric lymph nodes and pancreas.

**Figure 3:**
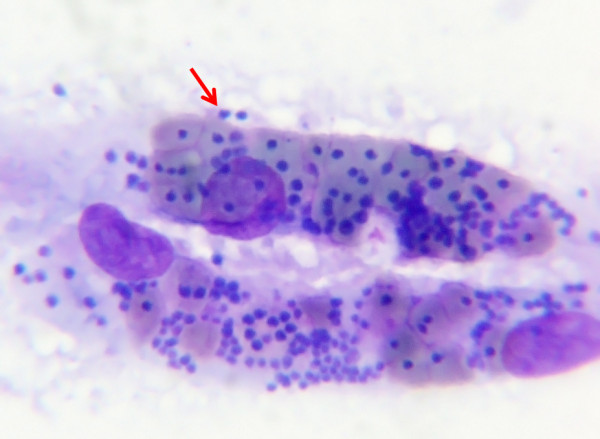
**Brain smear (Diff Quick stain) (Case 2): Cerebral capillary sludging of red blood cells that are heavily parasitized by a large *Babesia *(x1000) The dark, purple-bluish spots seen on the photograph represent the parasite in the erythrocyte (Photograph: Tertius Gous).**

Examination of blood smears by the pathologist revealed a mild parasitaemia of piroplasm trophozoites in erythrocytes, but brain smears showed massive numbers of piroplasms in and outside of erythrocytes (Figure 3). The brain capillaries were packed with heavily parasitised erythrocytes that resulted in sludging. The lesions and parasites in the brain were confirmed histopathologically.

### Confirmation of the presence of parasite DNA using partial 18S rRNA gene sequencing and phylogenetic analysis

DNA was extracted from 0.2 g pooled spleen and lymph node from Case 1 (BF226) and from 0.2 g brain tissue from Case 2 (BF463), using the commercially available QIAamp® DNA Mini Kit (Qiagen, Whitehead Scientific, South Africa), according to the manufacturer’s instructions. The V4 hypervariable region of the parasite 18S rRNA gene was amplified and screened using the reverse line blot (RLB) hybridization assay [22,23]. PCR products were subsequently sequenced (Inqaba Biotechnical Industries (Pty) Ltd, Pretoria, South Africa) using primers RLB-F and RLB-R [23].

The obtained sequence data were assembled and edited using GAP 4 of the Staden package (Version 1.6.0 for Windows) [24]. A search for homologous sequences was performed using BLASTn (http://www.ncbi.nlm.nih.gov) and sequences of closely related *Babesia* species were aligned with the obtained parasite sequences using ClustalX (Version 1.81 for Windows). The alignment was manually truncated to the size of the smallest sequence (405 bp). A phylogenetic tree was constructed using the neighbor-joining method [25] in combination with the boot-strap method (1000 replicates/tree) [26]. The consensus tree was edited using MEGA v4.0.2 [27].

**Figure 4:**
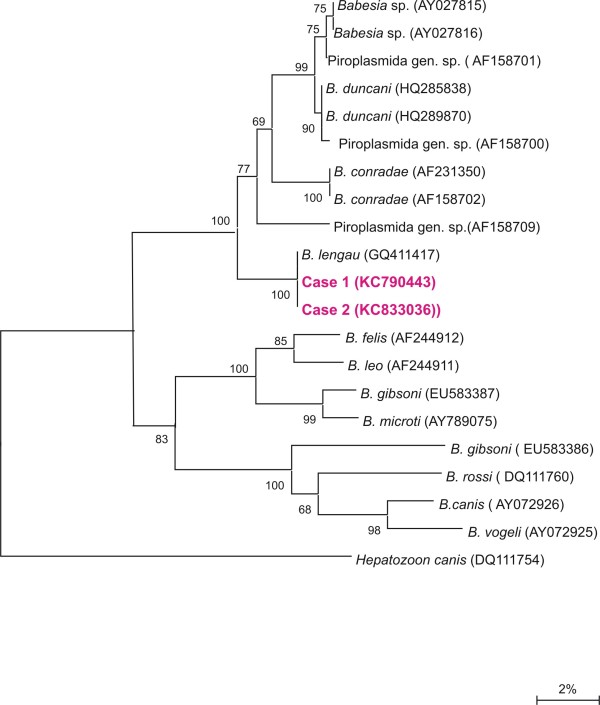
**Results of the neighbor-joining analysis of the 18S rRNA gene of the two piroplasms: The phylogenetic relationship of the piroplasms involved in cases 1 and 2 with other *Babesia*and *Theileria *species is shown.** The scale bar represents the % nucleotide differences.

Reverse line blot results from Case 1 (BF226) and Case 2 (BF463) showed positive hybridization profiles with the *Babesia lengau* species-specific probe [21]. The sequence data revealed that the two 18S rDNA sequences (KC790443 and KC833036) obtained (405 bp) were identical. A BLASTn search showed a high similarity (99.9%) with *B. lengau* (accession nrs GQ411405 to GQ411417) [21]; it differed by one deletion from *B. lengau*. A representative tree constructed by the neighbor-joining method using the two-parameter model of Kimura [28] is shown in Figure 4. Case 1 (BF226), Case 2 (BF463) and *B. lengau* (GQ411417) formed a monophyletic group with *B. conradae* (AF158702, AF231350), *B. duncani* (HQ285838, HQ289870) and other sequences previously identified from humans and wildlife in the western USA (AY027815, AY027816, AF158700, AF158701, AF158709) (Figure 4).

## Discussion

Both cases reported here differed in some respects from the usual clinical signs associated with feline babesiosis in South Africa [14]. Feline babesiosis is usually afebrile, while the body temperature of both cases was above the normal range when they were presented for treatment. Haemolytic anaemia, seen in Case 1, is not usually associated with typical feline babesiosis [14]. A *B. lengau*-like organism has recently been incriminated in causing a haemolytic disease in sheep in Greece [29].

Until recently, all small piroplasms of domestic cats that morphologically resembled *B. felis* were assumed to belong to that species. It has now been demonstrated that various small *Babesia* spp. can infect domestic cats [19]. Microscopic identification of piroplasms on blood smears is routinely done by practitioners to diagnose babesiosis in cats and other domestic animals. Confirming species identity, however, often requires molecular diagnostic tools. The reverse line blot hybridization assay and phylogenetic analysis are also more sensitive than blood smear examination in demonstrating the presence of *Babesia* spp. in subclinically infected animals.

The macroscopic and histopathological findings of both cases reported here suggested severe illness due to infection by a *Babesia* parasite, as described for dogs [30]. Molecular diagnostic methods confirmed the presence of *Babesia* parasites in blood and brain smears. Phylogenetic analysis revealed a high similarity to *B. lengau.*

*Babesia lengau* was described in asymptomatic cheetahs [21] and to date no evidence existed to suggest that *B. lengau* causes clinical disease in that host. The only other piroplasm *B. lengau* genotypically relates to closely is a canine parasite, *B. conradae*, which was described from dogs in California, USA, and is associated with haemolytic anaemia in the host [31,32]. *Babesia lengau* and *B. conradae* form part of the previously described “western clade” of piroplasms, which also includes *B. duncani* and piroplasms isolated from both humans and wildlife from the western USA. It clustered separately from the *Babesia* sensu stricto, the *B. microti* clade and *Theileria* and *Cytauxzoon* species.

Cerebral babesiosis has frequently been reported in cattle (e.g., caused by *B. bovis*) [33] and dogs (e.g., caused by *B. rossi*) [34,35]. To our knowledge, this serves as a first report of cerebral babesiosis in a domestic cat.

## Conclusion

All clinical cases of feline babesiosis in South Africa are not necessarily caused by *B. felis*. Other *Babesia* species, e.g. *B. lengau*, may be incriminated in clinical cases, especially those occurring outside the known endemic area. As a routine, the identity of the *Babesia* species involved should ideally be confirmed by molecular techniques when specimens from suspected feline babesiosis cases are submitted to diagnostic laboratories. Examining brain smears or sections is also recommended. Since the vector of *B. felis* has not yet been confirmed, our findings emphasise the urgency of further investigations to enhance understanding the epidemiology of this enigmatic disease.

## Competing interests

The authors declare that they have no competing interests.

## Authors’ contributions

AMB carried out the molecular genetic studies, participated in the sequence alignment and wrote the first draft of the manuscript; MCO supervised the laboratory work and participated in the sequence alignment and construction of the phylogenetic trees; EHV co-supervised the laboratory work; JCAS and TAG performed the necropsies and histological diagnostic investigation on Cases 1 and 2, respectively; BLP coordinated the investigation, conducted literature searches and wrote the final version of the report. All authors read and approved the final manuscript.
